# Emotion recognition and social functioning in individuals with autism spectrum condition and intellectual disability

**DOI:** 10.1371/journal.pone.0300973

**Published:** 2024-03-21

**Authors:** Daniela Tamas, Nina Brkic Jovanovic, Stanka Stojkov, Danijela Cvijanović, Bozana Meinhardt–Injac

**Affiliations:** 1 Faculty of Medicine, University of Novi Sad, Novi Sad, Serbia; 2 Catholic University of Applied Sciences Berlin, Berlin, Germany; University of Catania Department of Educational Sciences: Universita degli Studi di Catania Dipartimento di Scienze della Formazione, ITALY

## Abstract

**Objective:**

Most previous studies have examined emotion recognition in autism spectrum condition (ASC) without intellectual disability (ID). However, ASC and ID co-occur to a high degree. The main aims of the study were to examine emotion recognition in individuals with ASC and co-occurring intellectual disability (ASC-ID) as compared to individuals with ID alone, and to investigate the relationship between emotion recognition and social functioning.

**Methods:**

The sample consisted of 30 adult participants with ASC-ID and a comparison group of 29 participants with ID. Emotion recognition was assessed by the facial emotions test, while. social functioning was assessed by the social responsiveness scale–second edition (SRS-2).

**Results:**

The accuracy of emotion recognition was significantly lower in individuals with ASC-ID compared to the control group with ID, especially when it came to identifying angry and fearful emotions. Participants with ASC-ID exhibited more pronounced difficulties in social functioning compared to those with ID, and there was a significant negative correlation between emotion recognition and social functioning. However, emotion recognition accounted for only 8% of the variability observed in social functioning.

**Conclusion:**

Our data indicate severe difficulties in the social-perceptual domain and in everyday social functioning in individuals with ASC-ID.

## Introduction

Autism spectrum condition (ASC) is a neurodevelopmental condition characterized by a wide range of challenges in social communication and interaction, difficulty understanding nonverbal communication, intense interests, and the presence of repetitive and stereotyped behaviors [1–3]. According to DSM-5 and ICD-11, difficulties in social communication and interaction are key diagnostic features of ASC [2, 3]. Individuals with ASC may exhibit reduced social responsiveness, difficulties in non-verbal social communication, and challenges in initiating, maintaining, and understanding reciprocal social interactions [4–7]. Difficulties in understanding nonverbal communication signals [8, 9] and recognizing emotions [10–15] have also been frequently reported.

Despite variations in the findings of individual studies, recent meta-analyses and reviews have shown that individuals with ASC tend to have challenges in accurately recognizing emotions and exhibit longer reaction times compared to non-autistic individuals, particularly for certain emotions such as anger, fear, and sadness [10–15]. For instance, Uljarevic and Hamilton [13] found that individuals with ASC struggle with recognizing emotions, with fear recognition being particularly difficult. Other studies suggest that individuals with ASC may have disproportionate difficulties in recognizing negative emotions like anger, fear, and sadness [16, 17]. While individuals with ASC generally show lower accuracy and slower reaction times in recognizing emotions from facial stimuli, it is important to note that there is significant variability among individuals with and without ASC [17].

Most psychological theories of ASC emphasize a link between the skill of recognizing emotions from facial expressions and difficulties in using, sharing, and responding to emotions in everyday social interactions [18–20]. The findings of a meta-analysis [21] support this association, suggesting that the ability to recognize facial expressions is an important social cognitive skill in ASC that is related to real-life social functioning. Altered processing of emotions may contribute to challenges in social interaction and communication in individuals with ASC [16, 21–25].

The findings of these studies indicate a general association between the ability to recognize emotional facial expressions and social functioning in everyday life. However, there are conflicting findings on this topic [23]. It is important to note that these studies specifically focused on autistic children and adolescents with typical intelligence [10]. It remains unclear whether these findings can be generalized to adults with ASC, especially when ASC is accompanied by intellectual disability. It is also important to recognize that ASC and intellectual disability often coexist, with approximately 37.9% of 8-year-olds with ASC also being classified as having intellectual disability, and an additional 23.5% falling within the borderline range [26]. Individuals with ASC and intellectual disability have different abilities and needs compared to those with ASC alone [27]. Individuals with ASC and typical intelligence may employ alternative strategies for recognizing emotions that may not be observed when ASC is accompanied by intellectual disability [28, 29].

For this reason, our study specifically focused on facial emotion recognition in individuals with co-occurring ASC and ID. The objective of our study was to examine the difficulties in emotion recognition among individuals with ASC-ID as compared to those with ID only. Additionally, our study contributes to the limited research on the relationship between emotion recognition and social functioning in the adult population with ASC-ID and ID. We hypothesized that individuals with ASC-ID would exhibit lower accuracy in emotion recognition and demonstrate reduced social responsiveness. Furthermore, based on previous research conducted with children and adolescents, we anticipated finding an association between emotion recognition and social responsiveness in our adult sample as well.

## Materials and methods

### Participants

The sample for this study consisted of 59 participants, including 30 individuals with ASC and co-occurring intellectual disability (27 male), as well as a comparison group of 29 individuals (18 male) with intellectual disability alone (ID). A Kolmogorov-Smirnov test was conducted, revealing no significant differences in gender distribution between the groups (*p* > .10). The age range of all participants was between 18 and 48 years old, and another Kolmogorov-Smirnov test showed no significant differences in age distribution between the groups (*p* > .10). All participants in the ASC-ID group met the criteria for the third level of autism according to the DSM-5 [2, 30]. Participants in the ASC group had a diagnosis of autism spectrum disorder and were intellectually disabled, with IQ scores ranging from 35 to 55. The ID group consisted of individuals diagnosed with moderate intellectual disability. The diagnostic information for ASC and ID was obtained prior to the study through formal evaluations conducted by medical doctors with specialized training in autism and intellectual disability. Due to the utilization of different assessment measures, specifically the presence of a diagnosis of moderate intellectual disability without available IQ scores in the ID group, it was not possible to conduct statistical testing for differences in IQ between the ASC-ID group and the ID group. All participants were living with their families and attending an adult daycare program that provided quality care, as well as social and recreational activities tailored to individual needs.

### Materials

The instruments we used in this research were the Facial Emotion Test [31] and the Social Responsiveness Scale-2 [32]. Before testing face recognition and social responsiveness, socio-demographic data were collected.

### Facial emotions test

The Faces Test [31] was used to measure ability in basic emotion recognition. The test consists of black-and-white images of basic emotions (happy, sad, angry, afraid, surprised, disgusted, and distressed) and complex mental states (scheming, guilt, thoughtful, admiring, quizzical, flirting, bored, interested, and arrogant). One male and one female actor displayed these emotions. In original study, the stimuli were presented as the whole face, the eyes alone or the mouth alone and responses were given as force choice of two emotional words. For each stimulus, the subject was asked to choose the word under each photo that best described what the person was thinking or feeling [31, 33].

For the purposes of the present study, only photographs of the four basic emotions displayed in the whole faces were used: happy, angry, sad, and afraid. The basic emotions were selected to reduce complexity of the task. Each subject was tested individually in a quiet room. In total, each participant was presented with 4 trials in randomized order.

The test was performed by placing photographs of the four basic emotions presented by one model in front of the participant, and they were asked to point to the picture where facial expressions are happy/sad, angry/afraid ("Show me where this girl is sad?). The positon of the stimuli was counterbalanced across participants and trials. If the participant pointed to the appropriate picture it was recorded as a correct answer. If an inadequate picture was selected/shown or if no answer was given after repeating the same question several times within 5 minutes, this was counted as an incorrect answer. No feedback was provided. This procedure was repeated for each emotion and each stimulus. No specific training was conducted prior to the facial emotions test. However, all study participants received social interventions as part of the adult day care program, which also included guidance on recognizing emotions using pictures or cartoons. Reliability of the face emotions test in our sample was good (Cronbach’s Alpha = .86.).

### The Social Responsiveness Scale 2 (SRS-2)

The Social Responsiveness Scale 2 (SRS-2) [32] was utilized to evaluate social functioning. This questionnaire consists of 65 items that assess symptoms associated with ASC, with higher scores indicating more pronounced autistic traits The measure evaluates the level of social challenges across the complete autism spectrum, encompassing a range from nonexistent to severe. Parents/caregivers are asked to rate the presence of symptoms they have observed over time and in various settings. The SRS-2 comprises five subscales: Social Awareness (AWR) (e.g., " Is aware of what others are thinking or feeling”), Social Cognition (COG) (i.e., “recognizes and appropriately responds to changes in other people`s tone of voice and facial expression”), Social Motivation (MOT) (i.e., Avoids starting social interactions with other adults), Social Communication (COMM) (i.e. Seems self-confident when interacting with others,) and Restricted Interests and Repetitive Behavior (RRB) (i.e., Has repetitive, odd behaviors). Each item is rated on a four-point Likert scale: 1 = not true, 2 = sometimes true, 3 = often true, 4 = almost always true. The sum of all items yields a total score (maximum 195). T-scores are interpreted as follows: within normal limits (≤ 59 T/ total raw score below 67), mild (60–65 T / total score between 68–84), moderate (66–75 T/ total score between 85–112), and severe (≥ 76 T/ total score above 113). Due to the lack of evidence for their use in clinical decision-making and the lack of data stability and other psychometric data, the use of the total score is warranted [32]. Reliability of the total score in our sample was good (Cronbach’s Alpha = .85.).

### Procedure

Participants were assessed while they were attending a daycare center. Prior to the study, caregivers provided written informed consent, and verbal consent was obtained from the participants themselves. The face emotions recognition test was conducted in a quiet room, ensuring privacy and minimizing distractions, with only the participant and the test administrator present. The facial expressions were displayed for a maximum of 5 minutes, and participants were encouraged to complete the task. Special education specialists who had been working with the participants in the daycare center for at least one year completed the Social Responsiveness Scale, rating the participants’ behavior over the past 6 months. Data collection for participants with ASC-ID occurred between September 1st and December 15th, 2019. The control group with ID was surveyed between August 20th and November 1st, 2022.

### Ethics statement

Prior to the study, all potential participants and parents/ caregivers were informed in writing and oral form about the study aims, methods, sources of funding, any possible conflicts of interest, and institutional affiliations of the researchers. Participation was strictly voluntary. According to the Declaration of Helsinki, written in formed consent was obtained from parents/ caregivers; from participants with ASC-ID and ID the consent was obtained oral due to limitation in reading and writing. The agreement was documented by adding a “+” sign to the written consent of parents/ caregivers of the participant. The procedure for the study was approved by the Ethics Committee of the Medical Faculty, University of Novi Sad.

### Statistical analysis

We aimed to investigate group differences in emotion recognition and SRS-2 score, as well as the correlation between these variables. Prior to conducting data analysis, we performed the Lilliefors test to assess the assumption of normality for the mean score of emotion recognition and SRS-2 mean score. The results revealed a violation of the normal distribution assumption for both variables (Kolmogorov-Smirnov d = .22320, *p* < .01; Lilliefors *p* < .01 for emotion recognition; Kolmogorov-Smirnov d = .13, *p*>.20; Lilliefors p < .01 for SRS-2 mean score).The results indicated that both variables did not follow a normal distribution (Kolmogorov-Smirnov *d* = .22320, *p* < .01; Lilliefors *p* < .01 for emotion recognition; Kolmogorov-Smirnov *d* = .13, *p*> .20; Lilliefors *p* < .01 for SRS-2 mean score). Given the violation of the normal distribution assumption, we employed both parametric and nonparametric tests to assess group differences in emotion recognition and SRS-2 score. For example, we utilized the t-test and Mann-Whitney U Test, respectively. Additionally, we calculated correlation coefficients to explore the relationship between variables, as well as a linear regression model to investigate the potential contribution of emotion recognition to social functioning within our sample. The dataset used for this analysis is accessible at: https://doi.org/10.17605/OSF.IO/J8XM6.

## Results

### Group differences in accuracy of emotion recognition

**Fig 1:**
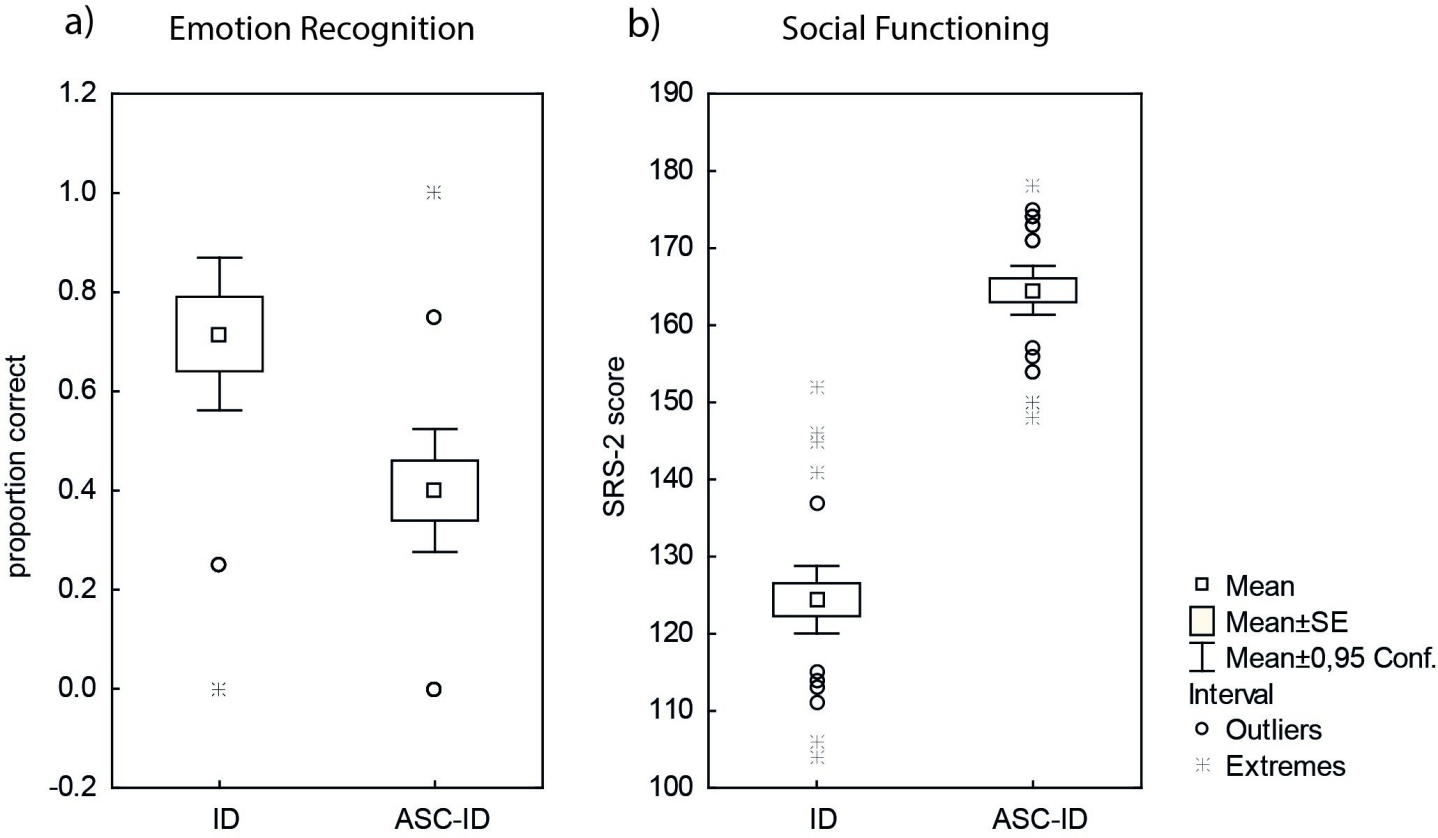
Results. a) Comparison of basic emotion recognition between individuals with Autism Spectrum Condition co-occurring with intellectual disability (ASC-ID) and a control group with intellectual disability only (ID). Individuals with ASC-ID demonstrated lower accuracy in emotion recognition, with statistical significance. b) Comparison of group differences in social functioning. Scores on the SRS-2 scale reflect the severity of social deficits and autistic traits, with higher scores indicating more pronounced difficulties in social functioning.

To examine whether groups with ASC-ID and a comparison group with ID were different in the accuracy of emotion recognition, we first calculated the proportion of correct responses as a mean for all four emotional states and each subject. First, a *t*-test for independent groups was applied to test differences in the accuracy of emotion recognition between ASC-ID and ID groups. The test was statistically significant, *t*(57) = 3.27, *p* < .002, with a medium effect size *r* = .39 [34], suggesting lower accuracy of emotion recognition in ASC-ID than in the ID group (Fig 1A). The same effects were obtained in the Mann-Whitney U Test for nonparametric data: *U* = 233.5, *p* = .002, with a medium effect size *r* = .39.

Next, we used ANOVA and Kruskal-Wallis-ANOVA to analyze differences in each of the four emotions (Table 1). ANOVA revealed significant main effects of emotion (*F*(3, 171) = 22.65, *p* < .001, and the group (*F*(1,57) = 5.87, *p* < .002), as well as significant emotion x group interaction (*F*(3,171) = 11.01, *p* < .001). There were no significant differences in recognition of happy (*F*(1, 57) = 1.17, *p* = .2) and sad (*H*(1, 57) = .54, *p* = .46) emotional expression, but ASC-ID individuals were significantly affected in recognition of angry (*F*(1, 57) = 18.3, *p* < .001, effect size *r* = .48) and afraid (*F*(1, 57) = 28.0 *p* < .001, effect size *r* =. 56) facial expressions. The effect size for both emotions (anger, fear) was strong [34]. The same effects were obtained when Kruskal-Wallis-ANOVA was applied.

**Table 1:** Emotion recognition accuracy.

	ASC-ID		ID
		*Mean (SD)*	*Mean (SD)*
**Happy**		.66 (.47)	.79 (.41)
**Sad**		.63 (.49)	.72 (.45)
**Angry**		.20 (.40)	.68 (.47)
**Afraid**		.10 (.30)	.65 (.48)
**ER Total**		.40 (.33)	.71 (.40)

Mean emotion recognition scores for four basic emotions (happy, sad, angry, and afraid) in the ID and ASC-ID groups. ER Total represents the average emotion recognition score across all emotions for each group. SD = standard deviation.

### Group differences in social functioning

To examine whether there were differences in social responsiveness, we first calculated a total score for each participant in the SRS-2 questionnaire. A T-Test for independent groups was applied to test differences in total score in SRS-2 (Fig 1B). The test was statistically significant, *t*(1,57) = -15.26, *p* < .001, with a strong effect size *r* = .89). ASC-ID group had significantly higher scores than ID group in SRS-. That indicate more severe difficulties in social functioning of ASC-ID group (Fig 1B). Again, the same effects were obtained when using Mann-Whitney U Test for non-parametric data.

### Correlation between emotion recognition and social functioning

To investigate the relationship between emotion recognition and social functioning in everyday life, we conducted a Pearson Correlation analysis between emotion recognition and the SRS-2 score. The results revealed a significant medium negative correlation: *r*(59) = -.32, *p* < .05. This suggests that lower emotion recognition abilities were associated with more difficulties in social functioning. Additionally, negative correlations were found between the SRS-2 score and the recognition of anger (*r*(59) = -.45, *p* < .001) and fear (*r*(59) = -.45, *p* < .001) emotional expressions. There were no significant correlations with the recognition of happy (*r*(59) = -0.12, *p* = .36) and sad (*r*(59) = -.05, *p* = .39) emotional expressions. However, when the correlations were calculated separately for the ASC-ID and ID samples, none of the correlations reached statistical significance (all *p* > .20). The Spearman rank-order correlation test yielded consistent results with these findings.

To assess the proportion of variance in social functioning that emotion recognition potentially explains, we conducted a linear regression analysis. The model including only the emotion recognition score as a predictor was statistically significant but accounted for only 8% of the variance in the SRS-2 Score (*t*(57) = 32.02, *p* < .001). In contrast, when the diagnosis of ASC-ID vs. ID was included as a predictor, it explained approximately 80% of the variance in the SRS-2 Score (*t*(56) = 14.53, *p* < .001).

## Discussion

This study aimed to investigate the ability to recognize facial emotions in individuals with ASC-ID and explore the association between emotion recognition and daily social functioning. While psychological theories of ASC [18–20] emphasize the connection between emotion recognition from facial expressions and social functioning, there is limited empirical research on this relationship [21]. Moreover, previous studies have primarily focused on children and adolescents with ASC and typical intelligence [16, 21–25]. It remains uncertain how applicable these findings are to other populations, such as adults with ASC, as emotion recognition abilities may undergo developmental changes, potentially leading to greater differences in adulthood compared to childhood and adolescence [10, 22].

The present study assessed emotion recognition from facial expressions [31] and daily social functioning [32] in a sample of 30 participants with ASC-ID and 29 participants with ID. The findings revealed general difficulties in facial emotion recognition among the ASC-ID group compared to the ID group, particularly in recognizing anger and fear. Both groups exhibited significant challenges in social functioning, with the ASC-ID group experiencing more pronounced difficulties. A negative correlation was observed between emotion recognition and social functioning, indicating that lower emotion recognition ability was associated with higher SRS-2 scores and greater difficulties in social functioning. However, it is important to note that emotion recognition explained only 8% of the individual variability in social functioning, while the diagnosis alone accounted for 80% of the variance. The results are discussed in more detail below.

### Basic emotion recognition in ASC

There is a significant body of research examining difficulties in recognizing emotions from facial expressions in individuals with ASC. These findings have been extensively analyzed in several meta-analytic studies [10–15]. A recent meta-analysis by Leung et al. [10] found consistent deficits in emotion recognition among individuals with ASC and typical intelligence (IQ > 70) compared to non-autistic individuals. These deficits were observed across all six basic emotions when recognizing facial expressions, while recognition of emotions from auditory cues, such as speech prosody, was selectively impaired for certain emotions (e.g., anger, joy, disgust). Trevisan et al [21] have shown that ASC-comparison group differences in emotion recognition were moderated, among others, by intellectual functioning of the ASC participants. A possible explanation is that persons with higher IQ and larger number of accumulated life experiences are better able to recognize and produce facial expressions that are more consistent with “non-autistic” norms [21, 35].

In line with these findings, our study also revealed general challenges in emotion recognition among the ASC-ID sample, with particularly pronounced difficulties in recognizing angry and fearful facial expressions compared to happy and sad expressions. Similar results have been reported in other studies, specifically regarding the recognition of negative emotions such as anger, fear, and sadness [16, 17]. There are several theoretical accounts for positivity bias in emotion recognition of the ASC individuals.

The amygdala theory of autism proposes that difficulties in recognizing negative emotions may be linked to dysfunction in the amygdala [33]. The amygdala plays a crucial role in processing social stimuli and it is highly sensitive to detecting and responding to threat-related information, including facial expressions of fear [36, 37]. Individuals with ASC exhibit deficits in amygdala activity when performing tasks related to processing facial expressions [38–40]. Additionally, several brain imaging studies have reported structural abnormalities in the amygdala of individuals with ASC [41]. However, the findings thus far have been inconsistent [42]. Alternatively, there is also the ’eye avoidance hypothesis’. According to the ’eye avoidance hypothesis’, individuals with ASC may avoid looking at the eye region of the face because they perceive it as socially threatening [43, 44]. It is well-established that different parts of the face, such as the eyes and the mouth, play varying roles in the recognition of facial emotions. For instance, research by Calder et al. [45] has shown that facial action units [46] involved in recognizing anger and fear are primarily located in the upper part of the face, while recognizing a happy facial expression relies more on the lower part of the face, specifically the mouth. Studies utilizing eye-tracking technology have provided further support for the notion of altered social attention and atypical gaze fixation in individuals with ASC during emotion recognition [47–49]. These differences in social attention and gaze behavior may help explain the variations observed in the recognition of happy versus fearful/angry emotions in the current study.

### Social functioning in ASC and emotion recognition

Difficulties in everyday social interactions are fundamental features of Autism Spectrum Condition (ASC). A meta-analysis conducted by Trevisan and Birmingham [21] indicates that the ability to recognize facial expressions plays a significant role in real-world social functioning. However, there is limited empirical research on this relationship within the ASC population, and further investigation is required to fully comprehend the significance of facial emotion recognition in the broader social challenges associated with ASC [21]. The main objective of this study was to explore the relationship between social functioning and the capacity to identify emotions.

In the existing literature, researchers have assessed the social functioning of individuals with ASC by employing measures of adaptive functioning and ASC symptomatology. For instance, Wallace et al. [16] investigated the relationship between the ability to recognize facial expressions and social functioning in adolescents with ASC who have typical cognitive abilities (IQ > 80), as well as a group of typically developing adolescents (TD). Social functioning was evaluated using the Autism Diagnostic Observation Schedule (ADOS), the Social Responsiveness Scale (SRS), and the Adaptive Behavior Assessment System-II (ABAS-II). The findings revealed that adolescence with ASC exhibited lower overall perceptual sensitivity to facial emotions compared to their typically developing counterparts. Additionally, all three measures of social and adaptive functioning (ADOS, SRS, ABAS-II) were linked to reduced perceptual sensitivity specifically towards sad facial expressions.

Bal et al [24] tested typically developing children and adolescents [7–17 years] and an age- and IQ-matched group with ASC in emotion recognition. The Social Responsiveness Scale (SRS) was used as a measure of social/emotional behaviour. Group differences in emotion recognition were found in latency, with the ASD group being significantly slower in emotion recognition. On measures of accuracy, group differences were found for anger, with the ASD group being less accurate than the control group. The severity of social deficits as measured by the SRS, were significantly correlated with accuracy in anger recognition, but there was no correlation with latency in emotion recognition.

In line with these previous studies, we evaluated social functioning using the Social Responsiveness Scale (SRS) [32]. Both groups demonstrated significant social difficulties, with ASC-ID individuals experiencing more pronounced difficulties (mean score: 164, range: 148–175) across all areas of social functioning when compared to the ID group (mean score: 124, range: 104–152). These findings highlight variations in the severity of social difficulties between the ASC-ID and ID groups. Most importantly, the current study revealed a significant correlation between emotion recognition score and the SRS-2 score (*r* = -0.33). Individuals with lower accuracy in emotion recognition exhibited more pronounced social challenges, as indicated by higher SRS-2 scores. However, when conducting separate statistical analyses for the ASC-ID and ID subgroups, no significant correlation was observed. Moreover, the ability to recognize emotions was found to account for only 8% of the variability in social functioning, whereas the diagnosis itself (ASC-ID vs. ID) explained 80% of the individual differences. These findings suggest an association between emotion recognition and social functioning, but they also highlight that emotion recognition is not the primary factor determining social challenges in the ASC population. It is important to interpret these results cautiously. These findings align with recent research indicating that the performance of ASC adults on standardized assessments of social cognition and social skills does not strongly predict their functional outcomes [50–53]. In fact, general cognitive abilities may have a stronger predictive value for social skills in autistic adults without intellectual disability compared to social cognition [53]. Therefore, explicit measures of social perception (such as emotion recognition) and social cognition may have limited predictive power for social communication and interaction behaviors in ASC.

### Limitations

This study is subject to several limitations that should be noted. First, although the number of trials and emotional stimuli used was relatively low, the study still yielded significant findings, indicating that the stimuli used were sufficient to detect differences in emotion recognition and social functioning. While increasing the number and variety of trials and stimuli may enhance the robustness and generalizability of future studies, the current findings provide a valuable starting point and direction for such future research. Second, the stimuli used in this study may not fully reflect real-world scenarios. Therefore, the use of more ecologically valid stimuli, which more closely mimic real-life situations, is recommended in future studies to ensure the findings are applicable to everyday life. Finally, although the lack of IQ scores for the ID group prevented a direct comparison of IQ between the two groups, it is noteworthy that the current study focused on emotion recognition and social functioning, rather than cognitive abilities. While it is recognized that IQ can influence both emotion recognition and social functioning, the primary aim of this study was to explore the relationship between emotion recognition and social functioning, independent of cognitive abilities. This focus provides valuable insights into the specific challenges faced by individuals with ASC-ID, which can inform the development of targeted interventions. Future studies should aim to incorporate IQ data to further elucidate the interplay between cognitive abilities, emotion recognition, and social functioning.

## Conclusions

In conclusion, this study contributes to the existing literature on emotion recognition in ASC with co-occurring ID. Our findings reveal a significant association between emotion recognition and social functioning in individuals with ASD-ID and ID. However, it is important to recognize that emotion recognition alone does not fully account for the social challenges observed in this population. Further research is warranted to investigate additional factors that may contribute to social challenges in the ASD-ID population, also considering the developmental perspective and the importance of early diagnosis [54]. By gaining a deeper understanding of the complex interactions between emotion recognition, social functioning, and other relevant factors, we can refine intervention strategies and enhance the effectiveness of interventions aimed at improving social functioning and emotion recognition skills in individuals with ASD-ID [55].
